# The Structural, Biophysical, and Antigenic Characterization of the Goose Parvovirus Capsid

**DOI:** 10.3390/microorganisms13010080

**Published:** 2025-01-03

**Authors:** Korosh Jabbari, Mario Mietzsch, Jane Hsi, Paul Chipman, Jianming Qiu, Robert McKenna

**Affiliations:** 1 Department of Biochemistry and Molecular Biology, Center for Structural Biology, McKnight Brain Institute, College of Medicine, University of Florida, Gainesville, FL 32610, USA; kjabbari1@ufl.edu (K.J.); janehsi@ufl.edu (J.H.); pchipman@ufl.edu (P.C.); 2 Department of Microbiology, Molecular Genetics and Immunology, University of Kansas Medical Center, Kansas City, KS 66103, USA; jqiu@kumc.edu

**Keywords:** goose parvovirus, cryo-EM, VLPs, capsid, three-dimensional structure, Muscovy duck parvovirus, variable regions, antigenicity, AlphaFold

## Abstract

Goose parvovirus (GPV) is an etiological agent of Derzsy’s disease, afflicting geese and Muscovy ducks worldwide. Its high mortality rate among goslings and ducklings causes large losses to the waterfowl industry. Toward molecular and structural characterization, virus-like particles (VLPs) of GPV were produced, and the capsid structure was determined by cryogenic electron microscopy (cryo-EM) at a resolution of 2.4 Å. The capsid exhibited structural features conserved among parvoviruses, including surface two-fold depressions, three-fold protrusions, and five-fold channels. A structural comparison of the GPV viral protein (VP) structure with other adeno-associated viruses (AAVs), including human AAV2, AAV5, and quail AAV (QAAV), revealed unique conformations of several surface-accessible variable regions (VRs). Furthermore, the GPV capsid was found to be thermally stable at physiological pH, but less so under lower pH conditions. As a member of the genus *Dependoparvovirus*, GPV could also be bound by cross-reactive anti-AAV capsid antibodies that bind to the five-fold region of the viruses, as shown by native immuno-dot blot analysis. Finally, the GPV VP structure was compared to those of other bird dependoparvoviruses, which revealed that VR-III may be important for GPV and Muscovy duck parvovirus (MDPV) infection.

## 1. Introduction

Goose parvovirus (GPV) is a member of *Parvoviridae*, a family of non-enveloped single-stranded DNA (ssDNA) viruses with T = 1 icosahedral capsids [1,2]. GPV belongs to the *Dependoparvovirus* genus, which mostly consists of non-pathogenic viruses that require coinfection with helper viruses, such as adenoviruses, to carry out their replication cycle [3]. Adeno-associated viruses (AAVs) are also in this genus, which are widely used as vectors for gene therapy applications [4]. It is noteworthy to mention that few dependoparvoviruses, such as GPV and Muscovy duck parvovirus (MDPV), are pathogenic and do not require coinfection with a helper virus to replicate in host cells [5]. The 5.1 kb GPV genome has two open reading frames (ORF) with inverted terminal repeats (ITRs) at either end that serve as origins of replication [6]. The “left” ORF expresses the non-structural proteins, which possess endonuclease-, helicase-, and DNA-binding functions that are responsible for the rolling hairpin replication of the genome, as well as the packaging of genomes into the capsid [7,8]. On the other hand, the “right” ORF expresses the three capsid viral proteins (VPs), VP1 (81 kDa), VP2 (65 kDa), and VP3 (60 kDa), that assemble the icosahedral capsid, with VP3 being the major capsid protein [6].

Dependoparvoviruses have been identified in many vertebrates, including humans, non-human primates, pigs, rodents, bats, cattle, birds, goats, reptiles, and sea lions [9,10,11,12,13,14,15,16,17,18]. Previously, the capsid structures of many of these dependoparvoviruses were determined through X-ray crystallography and/or cryogenic electron microscopy (cryo-EM) [19,20,21]. These capsids possess morphological features observed across all parvoviruses, including surface two-fold depressions, three-fold protrusions, and five-fold channels [22]. The VP monomers share a conserved anti-parallel β-barrel motif consisting of eight strands (βB to βI), an additional βA-strand running anti-parallel to the βB-strand, and an alpha-helix (αA) between the βC- and βD-strands. The loops connecting the β-strands of the β-barrel motif are identified based on the specific strands they link (for instance, the DE loop connects the βD- and βE-strands) [22]. In addition, segments of these loops have been defined and are known as variable regions (VRs), which are characterized by their sequence and structural diversity among different parvovirus genera [23]. The structural arrangement of the VRs of VP monomers within the T = 1 capsid creates the surface morphologies of parvoviruses. For example, five DE loops related by the pentameric symmetry of five VPs form the elevated pore of the twelve T = 1 five-fold channels, which is surrounded by a depressed surface consisting of five HI loops [19].

The disease caused by GPV was characterized in the 1960s by Derzsy, who described the epidemiology, clinical signs, and pathology associated with the virus [24]. GPV has since shown to be the causative effect of Derzsy’s disease in geese and Muscovy ducks and has afflicted waterfowl farming in Asia and Europe [25]. Vulnerable goslings and Muscovy ducklings are most severely affected by the disease, exhibiting an up to 90–100% mortality rate during their first few weeks of age [26,27,28]. Acute symptoms of these afflicted young birds include oculonasal discharge, oral diphtheria/necrosis, prostration, anorexia, headshaking, and swollen/crusted eyelids [29]. Typical pathological conditions associated with the disease include pulmonary edema, pericarditis, perihepatitis, hepatic/myocardial lesions, and ascitic fluid accumulation in the abdomen [25]. The vertical transmission of GPV can occur between mother and egg, while horizontal transmission typically happens through contact between uninfected birds or eggs and the feces of infected birds [29].

Though no treatment is currently available for Derzsy’s disease, several vaccines have been developed, some of which are commercially available. For decades, live, attenuated GPV vaccines have been developed through extensive viral passaging in goose embryo fibroblasts or Muscovy duck eggs [30,31,32,33]. As such, several vaccines have been administered in several countries, including VG32/1, SYG61v, and 82-0321V [34,35]. Additionally, Cevac Deparmune K, a vaccine containing inactivated strains of MDPV and GPV, is also commercially available for immunization against Derzsy’s disease and Muscovy duck parvoviral disease [36]. Despite commercial vaccine availability, the waterfowl industry is still impacted by GPV infections, and therefore, other kinds of GPV immunizations have been explored, including virus-like particles (VLPs), DNA vaccines, and bacterial vectors [37,38,39].

In this study, a baculovirus expression system was used to produce VLPs of GPV, which were purified and analyzed by cryo-EM to produce a reconstructed 3D structure of the GPV capsid, at a resolution of 2.43 Å. The built GPV VP structure was compared to predicted AlphaFold models, showing a high degree of structural similarity and RMSDs of ~0.7 Å and ~0.8 A. Structural comparisons between the GPV VP structure and other AAV structures and other bird dependoparvoviruses revealed unique conformations of various VRs within the GPV capsid, including VR-III, VR-IV, VR-V, and VR-VII. As a bird virus, human antibodies were not expected to bind to the GPV capsid. However, native dot blots showed that antibodies binding to the five-fold region of AAVs could also cross-react with the GPV capsid. The thermal stability of the GPV capsid was also studied over a range of pHs, revealing that it retains considerable thermal integrity in acidic conditions and has a higher melting point (T_m_) than several AAV serotypes at physiological pH. Overall, this study serves as the foundation for an understanding of the structural characteristics, antigenicity, and thermal stability of GPV, as well as a precedent for the development of a GPV vaccine that could prevent the devastating losses caused by Derzsy’s disease.

## 2. Materials and Methods

### 2.1. Virus-like Particle Production and Purification

VLPs of GPV were produced using the Bac-to-Bac^®^ [Invitrogen™, Waltham, MA, USA] baculovirus expression system, which was conducted following the manufacturer’s manual [40,41]. The VP3 gene of GPV was PCR-amplified from a plasmid containing the *rep* and *cap* genes of GPV [8]. The subsequent PCR product and the pFastBac™ plasmid [Invitrogen™, Waltham, MA, USA] were then subjected to XbaI and BamHI [NEB, Ipswich, MA, USA] restriction enzyme digestion separately, and the resultant DNA fragments were ligated together using T4 DNA ligase [NEB, Ipswich, MA, USA]. Then, TOP10 cells [Thermo Fisher Scientific, Waltham, MA, USA] were transformed with the recombinant pFastBac™ plasmid [Invitrogen™, Waltham, MA, USA] for cloning, and the amplified product was isolated using a miniprep kit [Invitrogen™, Waltham, MA, USA]. To prepare for baculovirus expression in SF9 cells [ATCC, Manassas, VA, USA], DH10Bac™ *E. coli* cells [Invitrogen™, Waltham, MA, USA] were transformed with the amplified and recombinant pFastBac™ plasmid [Invitrogen™, Waltham, MA, USA]. This permitted the transposition of the VP3 gene into the *lacZ* gene of the bacmid. SF9 cells [ATCC, Manassas, VA, USA] suspended and grown in Sf-900™ II SFM media [Life Technologies, Carlsbad, CA, USA] were transfected with the recombinant bacmid to produce a baculovirus stock. The recombinant baculovirus stock was then isolated and amplified by infecting SF9 cells [ATCC, Manassas, VA, USA] with the virus using a multiplicity of infection (MOI) of 0.5. The concentrations of the viral stocks were measured using viral plaque assays. Once a viral stock with the proper concentration was obtained after multiple amplifications, SF9 cells [ATCC, Manassas, VA, USA] were infected with the recombinant baculovirus at an MOI of 5 and then harvested after 72 h of incubation. The cells were centrifuged at 400× *g* for 15 min at 4 °C. The supernatant was then transferred to a separate container and stirred with polyethylene glycol (PEG)-8000 (10% *w*/*v*) overnight. Cell pellets were resuspended in 1× TNTM buffer (25 mM Tris-HCl, pH 8.0, 100 mM NaCl, 0.2% Triton X-100, 2 mM MgCl_2_), and then subjected to 3 freeze/thaw cycles. The cell pellet suspension was treated with benzonase (125 U/mL) [MilliporeSigma, Burlington, MA, USA] and digested at 37 °C for 1 h. The cell pellet sample was clarified through multiple cycles of centrifugation at 6000× *g* for 10 min at 4 °C, and the supernatant was transferred to a new tube after each spin. After overnight stirring, the supernatant–PEG mixture was centrifuged at 14,000× *g* for 90 min at 4 °C, and the supernatant was discarded. The pellet was resuspended in 1× TNTM buffer and clarified by centrifugation at 3000× *g* for 10 min at 4 °C. Cell pellet and supernatant VLP samples were then pipetted onto 20% sucrose cushions (*w*/*v* in 1× TNTM buffer) that were ultracentrifuged at 45,000 rpm for 3 h at 4 °C, using a TI-70 rotor [Beckman Coulter, Brea, CA, USA]. The pellets were resuspended in 1× TNTM, loaded onto a 5–40% sucrose gradient (*w*/*v* in 1× TNTM), and ultracentrifuged at 35,000 rpm for 3 h at 4 °C, using an SW41 Ti rotor [Beckman Coulter, Brea, CA, USA]. Fractions were collected in Eppendorf tubes, and SDS-PAGE was used to verify the presence of VLPs. Fractions showing the expected molecular weight of VP3 (~60 kDa) were dialyzed in 1× TD buffer (25.9 mM KCl, 8.8 mM KH_2_PO_4_, 684.5 mM NaCl, 51.1 mM Na_2_HPO_4_, 5 mM MgCl_2_·6H_2_O) and concentrated to 2 mg/mL using an Amicon^®^ Ultra 0.5 mL centrifugal filter [Merck Millipore, Burlington, MA, USA] with a 100 kDa cut-off. The dialyzed VLP samples were analyzed by negative-stain electron microscopy (EM) to check for the presence of intact GPV capsids. This involved the glow-discharging of a carbon-coated copper EM grid [Electron Microscopy Sciences, Hatfield, PA, USA] and then incubating it with 5 μL of the VLP sample for one to five minutes. Next, the grid was washed with DI water for 15 s and dried. Then, the grid was stained with 2% uranyl acetate for 30 s and then gently dried again. A Tecnai G2 Spirit electron microscope with a Gatan 2K × 2K charged-coupled device (CCD) camera, [Gatan, Pleasanton, CA, USA] operating at 120 kV, was used for the negative-stain EM procedure, as described previously [42].

### 2.2. Cryo-EM Data Collection

Holey carbon grids were glow-discharged, and 5 μL of the dialyzed VLP sample was applied to each grid. The grids were vitrified by plunge-freezing in liquid ethane using a Vitrobot Mark 4 [FEI, Hillsboro, OR, USA], with conditions at 4 °C and 95% humidity. Micrographs were taken using a Tecnai G2 F20-TWIN transmission electron microscope [FEI, Hillsboro, OR, USA] set to 200 kV at a low electron dose of 20 e^−^/A^2^, coupled with a 16-megapixel CCD camera set at a magnification of 82,500-fold. The micrographs taken were used to assess vitrification quality and particle distribution. For high-resolution structure determination, the vitrified grids were imaged using a Titan Krios G3 electron microscope with a K3 detector [Thermo Fisher Scientific, Waltham, MA, USA], operated at the Stanford-SLAC Cryo-EM Center. The microscope was operated at 300 kV, using a dose of 50 e^−^/A^2^, collecting 50 frames per micrograph. The movie frames were aligned using MotionCor2, as described previously [43].

### 2.3. Particle Reconstruction

The cisTEM software (version 1.0.0) was utilized for reconstructing the 3D structure of the GPV capsid. Micrographs were imported, and the contrast transfer functions calculated were used to eliminate any poor-quality micrographs. Particles were selected automatically using a particle radius of 125 Å and classified into 50 groups during 2D classification. Ten percent of the chosen particles were used for an ab initio 3D reconstruction, which generated an initial low-resolution model. Next, the auto-refinement tool was used to align particles against the initial low-resolution model for data refinement and the creation of a final 3D structure of the GPV capsid. The final structure was sharpened by whitening the upper and lower bounds of the amplitude spectrum as well as applying a negative B-factor. A pre-cut-off factor of −90 Å^2^ and different post-cut-off B-factors of 0, 20, and 50 Å^2^ were applied. The resolution of the final 3D structure was measured using a Fourier shell correlation (FSC) threshold of 0.143.

### 2.4. Model Building and Structure Refinement

A model of the GPV monomer was built using AlphaFold (version 2.3 and 3), based on the amino acid sequence of the GPV VP3. Sixty copies of the predicted structure were merged to create an entire capsid using the VIPERdb^2^ database, as previously described [44]. The resultant 60mer model was fit into the electron map of the capsid (a map with a post-cut-off B-factor of 20 Å^2^) using UCSF Chimera (version 1.17.3) [45]. Before fitting, the ‘vop zflip #0’ command was applied to correct the handedness of the electron density map, and the voxel size was optimized to maximize the model’s correlation with the data. The fitted GPV model was exported to Coot, in which the program’s tools were used to manually fit the monomer model into its electron density map for structural refinement [46]. The GPV capsid structure was further refined using PHENIX software (version: 1.10.2155), which provided refinement statistics (Table 1).

### 2.5. Sequence and Structure Comparisons

Sequence identities between GPV (NCBI accession #: NP_043515), quail AAV (NP_852781), AAV2 (YP_680426), and AAV5 (YP_068409) were determined by inserting their VP1 amino acid sequences from their FASTA files into NCBI blast [47]. For the determination of their structural identities, monomers from the pdb files for the capsids of GPV, quail AAV (PDB ID: 8TEX), AAV2 (8FYW), and AAV5 (3NTT) were analyzed in Coot, and the SSM superpose tool was used to align their structures [46]. Structural identities were calculated based on the percentage of aligned alpha carbons with distances less than 2 Å. For structural comparison of the refined and predicted AlphaFold models of the GPV monomer, the structures were also SSM-superposed on Coot, and their overall RMSDs were calculated by Coot. The refined GPV monomer model was also SSM-superposed (Coot) against AlphaFold2.3 models of Muscovy duck parvovirus (NCBI accession #: YP_068411), quail AAV, bar-headed goose parvovirus (QKE54992), monk parakeet parvovirus (WBY51255), pacific black duck AAV (QMI57778), white-backed woodpecker parvovirus (QKE60687), and chicken AAV (ACU30842). Overall RMSDs among the bird viruses were found using the alignment plugin on PyMOL (version 2.5.7) [48], and their sequence identities were found using NCBI blast (version 2.16.0).

### 2.6. Biophysical Characterization of Capsid Using DSF

The thermal stability of the GPV capsid in different pH environments was analyzed using differential scanning fluorimetry (DSF). SYPRO Orange dye [Invitrogen™, Waltham, MA, USA] was added to the VLP samples, as the dye molecules fluoresce when they bind to the hydrophobic regions of unfolded proteins. The fluorescence was detected and measured using an MiQ2 Thermocycler (Bio-Rad, Hercules, CA, USA). Eight universal buffer solutions (20 mM HEPES, 20 mM MES, 20 mM NaAc, 150 mM NaCl, and 3.7 mM CaCl_2_ • 2H_2_O) were prepared, all of which had different pHs of 1, 2, 3, 4, 5, 6, 7, and 8. To prepare GPV VLP samples, 21.5 μL of universal buffer, 2.5 μL of 1% SYPRO Orange (1× TD used as solvent), and 1 μL of purified GPV VLPs (~2 mg/mL) were added to individual wells of a 96-well plate. Samples were prepared in triplets, as each set of three VLP samples was made using a universal buffer with a particular pH. The initial temperature of the running assay was 30 °C, and the temperature was gradually increased to 99 °C in increments of 0.5 °C. The rate of change of the raw fluorescence data was tabulated as -dRFU/dt vs. temperature. The y-values (-dRFU/dt) of the graph were normalized by dividing them by the lowest dRFU/dt value (most negative). The temperature at the peak of the normalized graph for each sample was recorded as the T_m_ of the capsid, which is the temperature at which half of all the protein is unfolded and bound to fluorescent dye. Three T_m_ values were recorded for each of the eight pH environments.

### 2.7. Native Immuno-Dot Blot Analysis

A native immune-dot blot analysis was conducted using the Minifold I System (Cytivia, Marlborough, MA, USA). Particles of either GPV, AAV5, or AAV9 were added to each row of blots on the nitrocellulose membranes that were soaked in 1× TD buffer. Three amounts of viral particles, 10^10^, 0.5 × 10^10^, and 0.25 × 10^10^, were added to each row of the dot blot. Viral samples to be analyzed with B1 binding were placed in a heating block set to 100 °C for 10 min. Membranes with attached viral particles were incubated in a blocking solution of 6% milk (1× PBS) for 2 h and then incubated in a 1:1000 diluted primary antibody (monoclonal antibody or B1) in a solution of 6% milk and 0.1% Tween-20 overnight. Next, the membranes were washed with a solution of 1× PBS and 0.1% Tween-20 three times, for five minutes each. Then, the membranes were incubated in a secondary antibody (diluted in 6% milk and 0.1% Tween-20) conjugated to horseradish peroxidase (HRP) for 1 h. For membranes incubated in monoclonal antibodies (mAbs), HRP-conjugated goat polyclonal antibodies that bind to human IgG were used as secondary antibodies (1:50,000 dilution). For membranes incubated in B1 antibodies, HRP-conjugated anti-mouse IgG antibodies were used for secondary antibody incubation (1:3000 dilution). The membranes then underwent three more 5 min washes with 1× PBS and 0.1% Tween-20 and then were transferred to a film cassette. A solution of a 1:1 mixture of HRP substrate peroxidase solution and luminol reagent (Sigma-Aldrich, St. Louis, MO, USA) was added to the membranes, after which chemiluminescence was captured on X-ray film that was then developed for imaging.

## 3. Results and Discussion

### 3.1. Producing VLPs of GPV and Determination of Its 3D Capsid Structure

**Figure 1:**
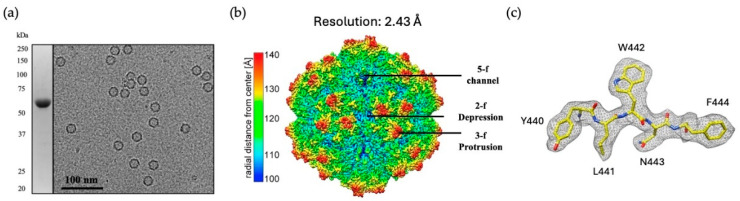
Determination of the GPV capsid structure. (**a**) SDS-PAGE of the GPV VLPs exhibiting the expected molecular weight of ~60 kDa and a cryo-EM micrograph of the same sample, displaying intact, empty capsids. (**b**) The 3D-reconstructed GPV capsid structure is shown with a final resolution of 2.43 Å. The approximate locations of surface features associated with the 2-, 3-, and 5-fold axes are indicated. The maps are colored according to the radial distance from the capsid center, as indicated by the scale bar. (**c**) A piece of the modeled βG-strand (aa440–aa444) of the GPV VP is shown inside the GPV electron density map. The model is displayed in a stick representation and colored according to atom type: C = yellow; O = red; N = blue.

The Bac-to-Bac^®^ baculovirus expression system was used to produce VLPs of GPV comprised of VP3 exclusively. While wild-type capsids contain approximately five copies of VP1 and VP2 each per capsid, previous studies have shown that VP3-only AAV capsids are structurally indistinguishable from wild-type capsids [49,50]. To determine its purity and concentration, the VLP sample was analyzed with SDS-PAGE. The only protein band observed possessed the expected molecular weight of VP3, which was approximately 60 kDa (Figure 1a). The concentration of the VLP sample was approximated to be 2 mg/mL, based on loaded bovine serum albumin (BSA) standards. The GPV capsids observed in cryo-EM micrographs had an estimated diameter of ~25 nm (Figure 1a). From these micrographs, capsid images were used to reconstruct a 3D GPV capsid structure with a final resolution of 2.43 Å (Figure 1b). Through the use of the electron density map, a model GPV VP monomer was built with a final map correlation coefficient of 0.844 (Figure 1c). The GPV capsid structure shows surface morphologies generally conserved among parvoviruses, including five-fold channels, three-fold protrusions, two-fold depressions, and two/three-fold walls. The five-fold channel connects the interior and exterior sides of the capsid and has been proposed to serve as a transit for the packaging or ejection of genomic DNA, as well as the externalization of the PLA_2_ domain of VP1u that is needed for the endosomal escape of parvoviruses during their endo/lysosomal trafficking following host cell entry [51,52].

**Figure 2:**
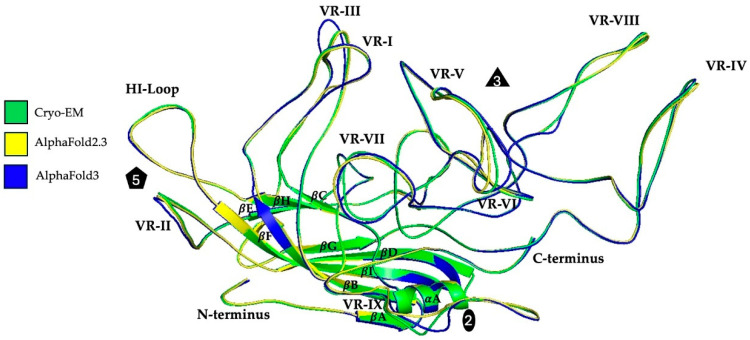
The GPV VP structure. The superposition of the experimentally determined (green) and AlphaFold2.3 (yellow)- and AlphaFold3 (blue)-predicted VP structures are shown as ribbon diagrams. The positions of VRs I to IX, the HI loop, β-strands (βA-βI), α-helix A, the N- and C-termini, and the icosahedral 2-, 3-, and 5-fold axes are indicated.

The high-resolution cryo-EM density map enabled the unbiased building of the GPV VP model. The beginning of structural ordering at the N-terminus was observed at alanine 214, which is similar to most AAV VP structures determined to date [21]. Further ordering may have been prevented possibly by the presence of a total of seven glycines following methionine 199, which is the VP3 start codon. Following alanine 214, the VP3 amino acid side chain densities were well ordered to leucine 732 at the C-terminus. The overall GPV VP structure conserves the core β-strand A, the eight-stranded anti-parallel β-barrel (βB-βI), and the α-helix A, as well as the general positioning of the surface loops (Figure 2). To test the prediction algorithm of AlphaFold (AF) (version 2.3 and 3.0) [53,54], GPV VP monomers were generated using its primary amino acid sequence and subsequently compared to the cryo-EM structure. The superposition of the highest-ranked AF models onto the experimentally determined structure resulted in Cα root mean standard deviations (RMSDs) of 0.69 and 0.78 Å, with most of the models in high agreement with the cryo-EM structure. However, structural deviations were observed in the VRs of the GPV monomer. Both AF algorithms had problems predicting VR-VII and VR-IX with local Cα-distances > 4 Å from the cryo-EM model. Similarly, VR-III and VR-V of the AF3 diverged by >5 Å, whereas the AF2.3 model performed better with these loops (Cα-distance < 2 Å) (Figure 2). Thus, in this case, the AF2.3 GPV model was overall slightly better than the model generated with the newer AF version. These deviations of the predicted models from the “real structure”, although small, may hinder future antiviral drug designs or receptor/antibody docking simulations, showing the need to determine the structure experimentally.

### 3.2. GPV Shows Structural Variation from the AAVs in Several Variable Regions

**Figure 3:**
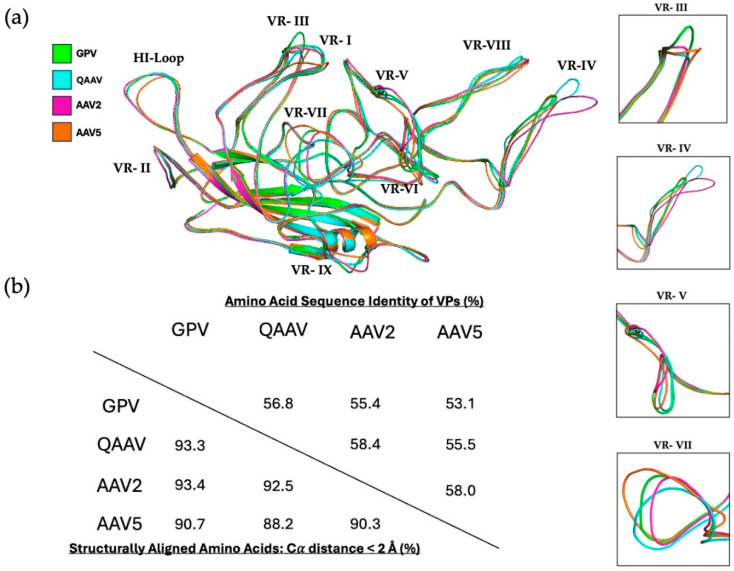
Sequence and structural comparisons of GPV (green), QAAV (turquoise), AAV2 (pink), and AAV5 (orange) monomers. (**a**) Alignment of the monomers of GPV, QAAV, AAV2, and AAV5. VRs containing significant structural variation between the monomers are boxed (right). (**b**) Table depicting the VP amino acid sequence and structural identities of GPV, QAAV, AAV2, and AAV5.

Since GPV is a member of the genus *Dependoparvovirus*, its VP structure was compared to the previously determined capsid structures of quail AAV (QAAV), AAV2, and AAV5 to identify the structural variation between these viruses (Figure 3a) [55,56,57]. Significant differences were observed in several variable regions, including VR-III, VR-IV, VR-V, and VR-VII. GPV has the largest VR-III loop, which is a result of AAV5, AAV2, and QAAV possessing one, two, or three amino acid deletions in the corresponding region, respectively. When compared to GPV, QAAV, AAV2, and AAV5 have local RMSDs of 1.36, 1.03, and 1.67 Å in VR-III, respectively. The VR-IV loops of GPV and AAV5 have very similar orientations, while those of AAV2 and QAAV are significantly larger. The local RMSDs of QAAV, AAV2, and AAV5 for this region are 5.04, 4.06, and 1.73 Å, respectively. The larger size and unique conformation of the VR-IV loop in the AAV2 capsid are responsible for its longer three-fold protrusions. Orientational variations in the VR-V and VR-VIII loops are also accountable for the morphological differences in the protrusions between the viruses. GPV and QAAV possess VR-V loops larger than those of AAV2 and AAV5, both of which have two deletions in the same region. The similar orientations of the VR-V loops of GPV and QAAV may be responsible for their smooth-edged and elliptical three-fold protrusions. The local RMSDs of VR-V are 1.51, 1.85, and 1.77 Å for QAAV, AAV2, and AAV5, respectively. Although all four viruses have VR-VI loops of the same size, the VR-VI region of AAV5 slopes more downward than those of the other viruses. The VR-VII loops of the viruses all possess unique conformations, with QAAV, AAV2, and AAV5 having local RMSDs of 2.23, 1.97, and 2.22 Å, respectively. To juxtapose the overall VP amino acid sequence and structural identities of the four viruses, a table showing the identities was created (Figure 3b). In spite of their low sequence identity, all four viruses share a large structural identity (Figure 3b). QAAV and AAV2 have the highest structural identities with GPV, both of which are greater than 93%.

All viruses showed conserved conformations in the VR-VIII region, a site important for AAV5 sialic acid (SIA) and AAV2 heparan sulfate proteoglycan (HSPG) receptor binding [58,59]. In a previous study, Afione et al. identified several residues in the VR-VIII region (residues 569–587) of AAV5 that interact with SIA [58]. The superimposition of the GPV and AAV5 monomers demonstrated that no residues of GPV match with those of AAV5 that are necessary for SIA interaction (only T578 of GPV matched with T571 of AAV5). Additionally, Opie et al. found that two basic residues (R585 and R588) in the VR-VIII loop of AAV2 are essential for HSPG interaction [59]. However, GPV does not possess either of these critical residues. Residues important for the AAV receptor (AAVR) binding of AAV2 and AAV5 were also compared with those of GPV. Zhang et al. collected cryo-EM data from samples of AAV2 or AAV5 complexed with AAVR and identified AAV residues constituting the virus–receptor interface. Multiple VRs of AAV2, such as VR-I, -III, -IV, -V, -VI and -VIII, mediate binding with the polycystic kidney disease (PKD) ectodomain 2 of AAVR (PKD2) [60]. Moreover, Zhang et al. also found residues of AAV5, largely from VR-VII and VR-IX, that interact with PKD1 of AAVR [61]. Residues of GPV aligned with those of AAV2 and AAV5 do not match any of the AAV residues that mediate AAVR binding. In short, GPV may not interact with SIA nor HSPG for transduction initiation, and it is unlikely that the GPV capsid binds to AAVR. Further study aimed at studying GPV and receptor interactions is needed to elucidate the mechanism by which GPV initiates transduction.

### 3.3. Cross-Reacting Human mAbs Bind to GPV in Its Five-Fold Region

**Figure 4:**
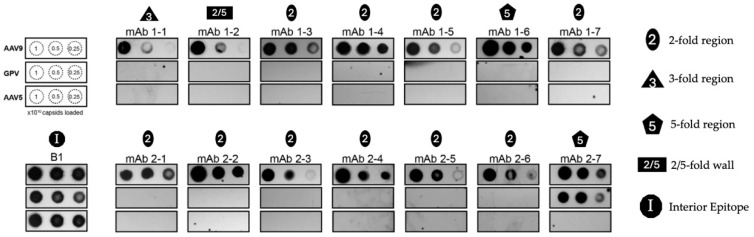
Native immuno-dot blot analysis of mAbs reconstituted from infant patients with SMA treated with Zolgensma, an AAV9 vector gene therapeutic. Decreasing amounts of wtAAV9 capsids, VLPs of GPV, or wtAAV5 capsids are loaded onto the membranes from left to right in each row of blots (upper-leftmost panel). The dot-blots from the mAb incubations are shown and labeled with the conserved morphological region that the mAbs bind to. The dot blot from the B1 antibody, which binds to a linear epitope buried in the interior of the capsid, serves as a loading control for the capsids (denatured, lower-leftmost panel) [67].

One of the most critical hurdles that undermine the efficacy of AAV gene therapy is the prevalence of neutralizing antibodies (NAbs) against different AAV serotypes in human populations. Antibodies against AAV1 and AAV2, for instance, are very prevalent (in up to 70% of the population), and patients seropositive for certain serotype-specific NAbs are rendered ineligible to receive AAV gene therapy in clinical trials [62,63]. The co-prevalence of different anti-AAV NAbs and their cross-reactivity with multiple different serotypes only compounds the issue of humoral immunity against AAV vectors [62,64]. As a bird-based virus, GPV may be able to escape the binding of anti-AAV NAbs. To test this theory, native immuno-dot blot analyses of the VLPs of GPV, wild-type AAV5, and wild-type AAV9 capsids were conducted using mAbs reconstituted from the switched memory B cells of two infant patients with SMA (patients 1 and 2) treated with Zolgensma, an AAV9-based gene therapy for treating the neuromuscular disorder (Figure 4) [65]. The AAV9 vector prompted an IgG immune response against its capsid, and seven different mAbs were isolated from each patient. Logan et al. created electron density maps of complexes between AAV9 and fragment antigen-binding (Fab) antibodies that demonstrate that the mAbs bind to the five-, three-, and two-fold regions of the capsid, as well as the two/five-fold walls. A dot blot analysis of the VLPs of GPV indicated that only mAb 2–7, which binds to the five-fold region, cross-reacts with GPV. The mAbs 1–1 and 1–2, which bind to the three-fold region and the two/five-fold wall, respectively, are unable to bind the GPV capsid. The rest of the mAbs (except mAb 1–6) bind to the two-fold region, and they are also unable to bind to the GPV capsid. Their inability to bind to the GPV capsid can be attributed to their orientational incompatibility with surface morphologies, since the two-fold and three-fold regions, as well as the two/five-fold walls, exhibit structural variability among different AAVs [19]. Logan et al. showed that mAbs 1–6 and 2–7 can cross-react with AAV1–9 serotypes (except AAV5), as well as with AAVrh.10, since they recognize the five-fold region that is strongly conserved among AAVs [65]. Monomer alignments of GPV, QAAV, AAV2, and AAV5 show that the DE and HI loops (which form the five-fold region) of GPV have very similar conformations, which explains its mAb 2–7 recognition (Figure 3a). To resolve the question of why mAb 1–6, which also binds to the five-fold region, was unable to recognize GPV, AAV9 residues that constitute the Fab 1–6 and Fab 2–7 interfaces were structurally aligned with the corresponding residues in GPV. Mietzsch et al. discovered these interface residues by building models of the Fabs and the AAV9 capsid, and then fitting them into a high-resolution electron density map produced from cryo-EM data [66]. The majority of the aligned GPV and AAV9 residues matched for both interfaces. A superimposition of the GPV and AAV9 monomers showed that the aligned residues of GPV had more similar orientations to AAV9 residues constituting the mAb 2–7 interface than the AAV9 residues that make up the mAb 1–6 interface. The calculated RMSDs of the aligned GPV and AAV9 residues for the mAb 2–7 and mAb 1–6 interfaces were 0.6 and 2.6 Å, respectively. This significant difference in structural similarity accounts for the GPV capsid binding discrepancy between mAbs 1–6 and 2–7. In short, GPV capsid regions that closely mimic AAV morphologies can be recognized by cross-reacting mAbs.

### 3.4. The GPV Capsid Has High Thermal Stability

**Figure 5:**
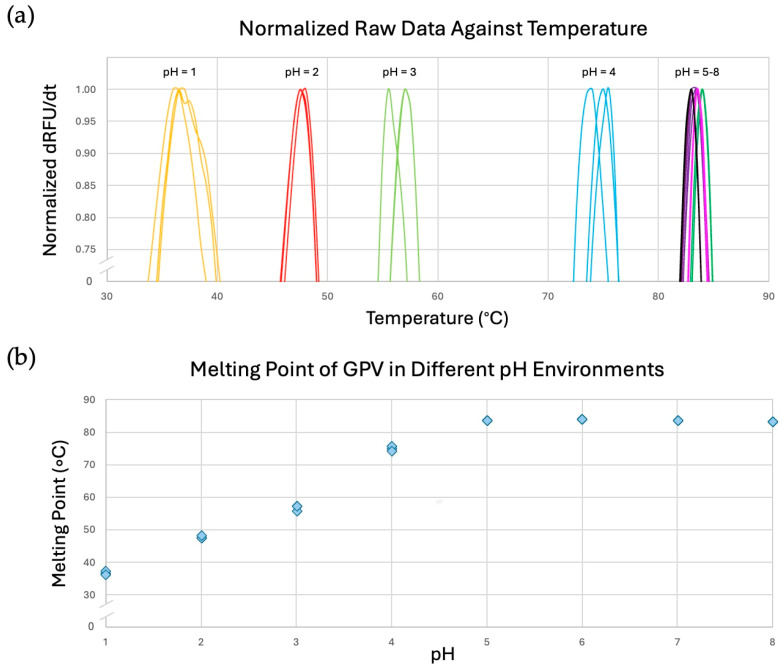
DSF data characterizing the thermal stability of the GPV capsid in different pH environments. (**a**) Raw fluorescence data curves from DSF of the VLPs of GPV at pHs of 1–8. Each color represents a discrete pH value. (**b**) Scatterplot of the measured melting points of the VLPs of GPV against pH. The experiments were conducted in triplicate.

AAVs have been biophysically characterized and identified using DSF, a procedure that analyzes protein thermal stability by measuring the fluorescence of dye molecules that bind to the hydrophobic interior regions of denaturing protein [68]. AAV capsids exhibit melting points that vary from 65° (AAV2) to 89 °C (AAV5) at physiological pH [69]. To determine the thermal stability of the GPV capsid, the VLPs of GPV were subjected to DSF using SYPRO Orange dye. Additionally, DSF assays of the VLPs were conducted in multiple different pH environments to analyze changes in the thermal stability of the capsid throughout the GI tracts of waterfowls. Different segments of the GI tracts of ducks have acidic environments that range from a pH of 2.3 in the gizzard to a pH of 6.9 in the lower ileum [70]. DSF of the VLPs was conducted in triplicate for each pH (pH 1–8) (Figure 5a). The T_m_ is defined as the temperature when 50% of all protein is denatured and bound to fluorescent dye molecules. Three melting points were recorded and averaged for each pH environment, and the averages were as follows: 36.5 °C at a pH of 1.0; 47.7 °C at a pH of 2.0; 56.5 °C at a pH of 3.0; 74.8 °C at a pH of 4.0; 83.5 °C at a pH of 5.0; 84 °C at a pH of 6.0; 83.5 °C at a pH of 7.0; and 83 °C at a pH of 8.0. To analyze how the T_m_ changes with respect to pH, the measured melting points from all 24 data curves were plotted against pH (Figure 5b). According to the trend of the scatterplot, the GPV capsid seems to exhibit higher thermal stability than the serotypes AAV2, AAV4, and AAV7–10 at physiological pH [69]. The melting point of the AAV3 capsid is like that of the GPV capsid at physiological pH. Only AAV5 (T_m_ of approximately 89 °C) clearly possesses a higher thermal stability than GPV at physiological pH [69]. Additionally, the GPV capsid has a melting point higher than the physiological body temperature of waterfowls (ranging from 39 °C to 43 °C) between pHs 2 and 3 [71]. This suggests that the GPV capsids remain intact inside the highly acidic environments of duck gizzards. The trendline of the scatterplot demonstrates that increasing the pH of the environment toward physiological pH favors the thermal stability of the GPV capsid.

### 3.5. Many Bird-Derived Dependoparvoviruses Are Structurally Similar to GPV

**Figure 6:**
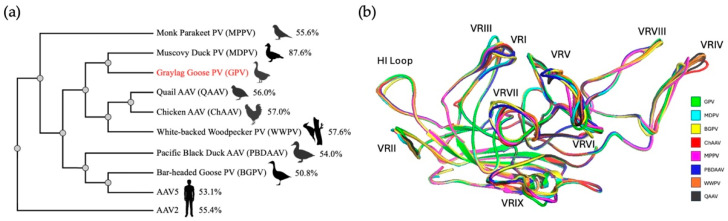
Comparisons of the VP amino acid sequences and structures of different bird-based dependoparvoviruses as well as AAV2 and AAV5. (**a**) Phylogenetic analysis of the VP amino acid sequences. Sequence identities compared to GPV are given for all viruses as a percentage. (**b**) Structural alignment of the predicted AlphaFold2.3 models of seven bird-based dependoparvoviruses with the cryo-EM GPV model. All the VRs, as well as the HI loop, are labeled.

The first bird-based dependoparvovirus was described in 1973 from bobwhite quail and was named avian AAV [72]. To date, several dependoparvoviruses have been isolated from different avian species. However, the names of these viruses have not been adjusted for the many newly identified members of the genus. Thus, for more specificity, the original avian AAV was renamed quail AAV (QAAV) in this study. To analyze the amino acid sequence variation of various bird-derived dependoparvoviruses, a phylogenetic tree of the VP sequences of GPV, MDPV, bar-headed goose parvovirus (BGPV), chicken AAV (ChAAV), monk parakeet parvovirus (MPPV), pacific black duck AAV (PBDAAV), white-backed woodpecker parvovirus (WWPV), and QAAV was generated (Figure 6a). Of note, MDPV is most similar to GPV, as they share a VP sequence identity of almost 88%, which has been stated previously [6]. The VP sequences for the other bird dependoparvoviruses significantly differ from GPV, with sequence identities ranging from 50.8 (BGPV) to 57.6% (WWPV). For further insight into the structural differences between these bird viruses, predicted models of the VP3 monomers of these viruses were generated through AlphaFold and aligned with the refined GPV monomer for comparison (Figure 6b). The alignment showed that conformational differences were localized to VR-I, VR-III, VR-IV, VR-V, VR-VII, and VR-IX. Overall, all predicted models of the bird-based dependoparvovirus monomers were structurally similar to the GPV monomer, with RMSDs ranging from 0.39 (MDPV) to 0.54 Å (BGPV). MDPV has, expectedly, the highest structural similarity to GPV, since it exhibits a significantly higher sequence identity than the other bird viruses. Interestingly, the alignment revealed that the VR-III regions of GPV and MDPV possess an identical size and conformation. This suggests that VR-III may play an important role in the infection cycle of both waterfowl viruses. BGPV and PBDAAV exhibit the highest overall structural differences from GPV, both of which have RMSDs of approximately 0.5 Å, which was expected since BGPV and PBDAAV also have the lowest VP amino acid sequence identities with GPV (which are 50.8% and 54%, respectively). Moreover, structural superimposition revealed that BGPV and PBDAAV have larger VR-VII regions with similar predicted orientations. This finding suggests that the conformation of VR-VII may have an important function in the infectivity of BGPV and PBDAAV. WWPV, QAAV, and CHAAV all exhibit larger VR-IV loops, with four amino acids inserted in the corresponding region compared to GPV. Lastly, MPPV has the smallest VR-V loop and the largest VR-IX loop, characterized by four amino acid deletions and four insertions (compared to all the other bird viruses), respectively.

## 4. Conclusions

GPV is an etiological agent of Derzsy’s disease, which has caused severe economic losses to the waterfowl industry, killing many goslings and Muscovy ducklings worldwide [25,27,28,73]. VLPs of GPV were expressed and purified, leading to a structural analysis of the particles using cryo-EM, producing a 3D structure of the capsid with a final resolution of 2.43 Å. The GPV capsid exhibited morphological surface features conserved among many parvoviruses, including three-fold protrusions, five-fold channels, and two-fold depressions [19,22]. The GPV VP exhibited the tertiary structural features typically found in parvoviruses, including the characteristic anti-parallel eight-stranded β-barrel [22]. The refined model of the GPV monomer was compared with those of QAAV, AAV2, and AAV5. The structural differences between the VPs were localized to VRs III, IV, V, and VII. The structural observations of GPV add to the repertoire of bird-based dependoparvovirus structures, which is currently limited to just QAAV [55]. This study may also serve as a foundation for the rational structural design of GPV capsid-based vaccine development to prevent the widespread effects of Derzsy’s disease. In addition, the native immuno-dot blot analysis of GPV VLPs with anti-AAV9 mAbs isolated from infant patients with SMA (treated with Zolgensma) [65] demonstrated that human-derived mAbs that bind to the five-fold region conserved among AAVs are also able to bind GPV, indicating that anti-AAV antibodies can cross-react with GPV, a bird-based parvovirus. The thermal stability of the GPV capsid was also characterized by a DSF assay. The data showed that GPV exhibits a higher thermal stability than many AAV serotypes at physiological pH, and that the capsid remains intact even in the acidic environments of the GI tracts of ducks [70]. Finally, the structural alignment of the GPV VP and predicted models of other bird-based dependoparvoviruses revealed high structural similarity for all the viruses, with MDPV possessing the highest structural similarity to GPV and both viruses having identical VR-III loops. Further functional structure-guided studies will be needed to determine if VR-III is crucial to the infection cycle of the two closely related waterfowl viruses.

## Figures and Tables

**Table 1:** Summary of the cryo-EM data collected and PHENIX refinement statistics.

Cryo-EM Data and Refinement Parameters	
Number of Micrographs	1716
Defocus Range (μm)	0.4–4.7
Electron Dose (e^−^/A^2^)	50
Frames/Micrograph	50
Pixel Size (Å/pixel)	0.82
Capsids Used for Final Map	15,471
Resolution of Final Map (Å)	2.43
**PHENIX Model Refinement Statistics**	
Map CC	0.844
RMSD Bonds (Å)	0.01
RMSD Angles (°)	0.86
All-Atom Clashscore	6.82
**Ramachandran Plot**	
Favored (%)	97.5
Allowed (%)	2.5
Outliers (%)	0
Rotamer Outliers (%)	0
C-β Deviations	0

## Data Availability

The GPV cryo-EM-reconstructed density map and model built for the capsid were deposited in the Electron Microscopy Data Bank (EMDB), with accession numbers EMD-48181 and PDB ID 9ME0.
